# Construction of a Visible-Light-Response Photocatalysis–Self-Fenton Degradation System of Coupling Industrial Waste Red Mud to Resorcinol–Formaldehyde Resin

**DOI:** 10.3390/molecules29071514

**Published:** 2024-03-28

**Authors:** Xiangxiu Lv, Hao Yuan, Kaiqu Sun, Weilong Shi, Chunsheng Li, Feng Guo

**Affiliations:** 1 School of Material Science and Engineering, Jiangsu University of Science and Technology, Zhenjiang 212100, China; 2 Key Laboratory of Advanced Electrode Materials for Novel Solar Cells for Petroleum and Chemical Industry of China, School of Chemistry and Life Sciences, Suzhou University of Science and Technology, Suzhou 215009, China; 3 School of Energy and Power, Jiangsu University of Science and Technology, Zhenjiang 212100, China

**Keywords:** photocatalysis–self-Fenton, in situ H_2_O_2_ production, red mud, antibiotic degradation, resorcinol–formaldehyde

## Abstract

Heterogeneous photocatalysis–self-Fenton technology is a sustainable strategy for treating organic pollutants in actual water bodies with high-fluent degradation and high mineralization capacity, overcoming the limitations of the safety risks caused by adding external iron sources and hazardous chemicals in the homogeneous Fenton reaction and injecting high-intensity energy fields in photo-Fenton reaction. Herein, a photo-self-Fenton system based on resorcinol–formaldehyde (RF) resin and red mud (RM) was established to generate hydrogen peroxide (H_2_O_2_) in situ and transform into hydroxy radical (^•^OH) for efficient degradation of tetracycline (TC) under visible light irradiation. The capturing experiments and electron spin resonance (ESR) confirmed that the hinge for the enhanced performance of this system is the superior H_2_O_2_ yield (499 μM) through the oxygen reduction process (ORR) of the two-step single-electron over the resin and the high concentration of ^•^OH due to activation effect of RM. In addition, the Fe^2+^/Fe^3+^ cycles are accelerated by photoelectrons to effectively initiate the photo-self-Fenton reaction. Finally, the possible degradation pathways were proposed via liquid chromatography-mass spectrometry (LC-MS). This study provides a new idea for environmental recovery in a waste-based heterogeneous photocatalytic self-Fenton system.

## 1. Introduction

The abuse of antibiotics (e.g., tetracycline) and the stability of their molecular structures lead to their usual detection in natural aquatic systems and causing ecotoxicological effects that have brought many adverse effects to the ecological environment, human health, microbial growth, etc. [1,2,3]. In order to solve the pollution problem of antibiotic wastewater, biological treatment, chemical oxidation, and physical adsorption methods are proposed, but they suffer from high energy consumption, high cost, complex operation, or secondary pollution [4,5,6,7]. Recently, as advanced oxidation processes (AOPs), the rapid expansion of Fe in a 2+ oxidation state (Fe^2+^) and the hydroxyl radical (^•^OH)-based Fenton oxidation method, since ^•^OH expresses high redox potential (+2.80 V vs. SHE), for organic matter decomposition [8,9,10] have been broadly applied in antibiotic wastewater treatment. Unfortunately, the conventional homogeneous Fenton method suffers from poor tolerance of pH, high difficulty in separating iron species from water and weak Fe^2+^ regeneration [11]. Furthermore, the continuous addition of consumable hazardous chemical oxidants (hydrogen peroxide, H_2_O_2_) significantly increases expenses and security risks during the process of production and transport, which seriously restricts its actual application in treatment of pollutant wastewater [12]. Accordingly, it is urgent to seek a sustainable method for overcoming the shortcomings of the homogeneous Fenton technique and improving its efficiency without any extra addition of H_2_O_2_.

**Scheme 1:**
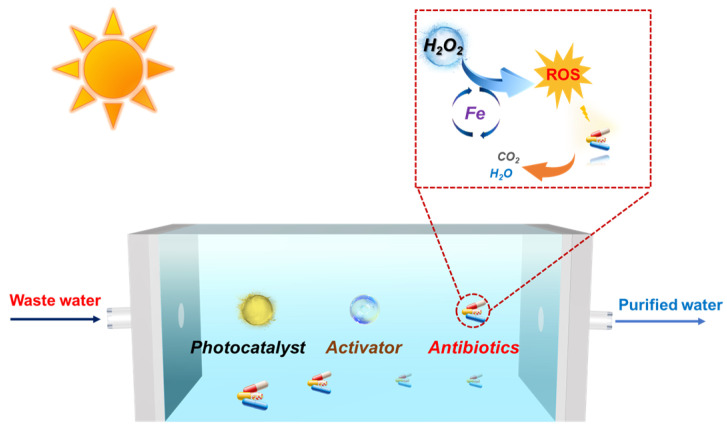
Antibiotic wastewater treatment via the photo-self-Fenton purification system.

Photo-driven heterogeneous photocatalysis–self-Fenton technology offers tremendous superiorities in terms of economic efficiency, environmental compatibility, security, and pH survivability in contrast to the conventional homogeneous Fenton process [13]. In this route, H_2_O_2_ can be synthesized in situ through the semiconductor photocatalysis without the need for external additions, effectively circumventing the risks associated with H_2_O_2_ storage and transport. Photocatalytic H_2_O_2_ production is generally considered a green and sustainable model owing to the boundless supply of solar energy [14,15]. Furthermore, H_2_O_2_ formed spontaneously would be activated by Fe^2+^ ions to produce ^•^OH (Fe^2+^ + H_2_O_2_ → Fe^3+^ + ^•^OH + OH^−^, *k* = 40–80 L∙mol^−1^ s^−1^); the produced Fe^3+^ was further reduced by photo-generated electrons, which leads to the continuous Fenton effect due to the effective acceleration of Fe^2+^/Fe^3+^ that recognized rate-limiting step in the Fenton reaction (Fe^3+^ + H_2_O_2_ → Fe^2+^ + ^•^OOH + H^+^, *k* = 0.001–0.01 L∙mol^−1^ s^−1^) [16,17]. In addition, the homogeneous Fenton system is limited by a narrow pH range (pH ≤ 3), and the liquid Fe-based activators are hardly separated and removed from the water body, causing secondary pollution of the purified water quality, which results in additional costs owing to the treatment of Fe mud pollution [18]. By comparison, heterogeneous photocatalysis–self-Fenton technology perfectly overcomes the shortcomings of pH tolerance, secondary pollution, and activator recycling, meeting the theoretical support for the practical application of real-time wastewater treatment in actual dynamic flow systems (Scheme 1) [19]. Accordingly, it is essential to explore a high-performance photocatalyst capable of generating H_2_O_2_ and a suitable Fe-based activator to construct a heterogeneous system due to the fundamental impact endowed by the production and activation of H_2_O_2_.

In this work, a heterogeneous photocatalysis–self-Fenton system was constructed by employing resorcinol–formaldehyde (RF) resin as photocatalyst and industrial waste red mud (RM) as activator. Resorcinol–formaldehyde resin, as a metal-free polymer photocatalyst, shares an absorption wavelength up to 700 nm and solar–chemical conversion efficiency of 0.5% due to a narrow band gap (2.0 eV), thus stably and effectively driving the photocatalytic in situ production of H_2_O_2_ from water [20]. Moreover, RM is a kind of solid iron source from industrial wastes [21], and introducing the cheap iron source as a key to initiate the self-Fenton process not only achieves the separation of iron ions from water, but also reduces the industrial cost and controls the environmental pollution due to industrial production, thus achieving the effect of killing three birds with one stone. Electron spin resonance (ESR) and liquid chromatography-mass spectrometry (LC-MS) were conducted to further discuss the mechanism and the intermediates for the RF-based photo-self-Fenton system in detail. This work presents an in-depth insight for designing a sustainable and economical heterogeneous self-Fenton system.

## 2. Results and Discussion

**Figure 1:**
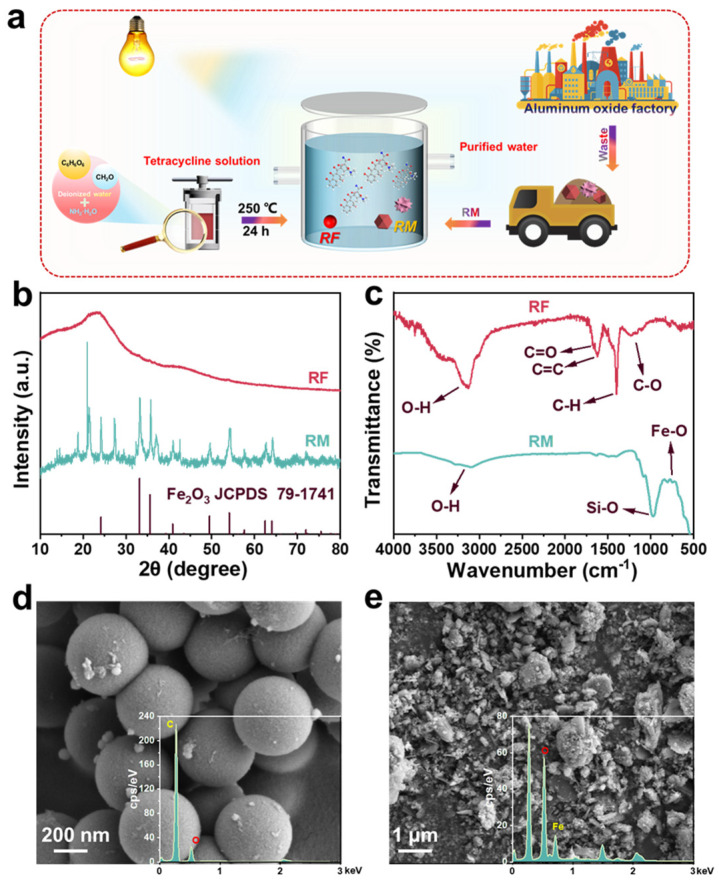
(**a**) Schematic diagram of RF/RM photo-self-Fenton degradation system. (**b**) XRD patterns and (**c**) FT-IR spectra, (**d**) SEM images, and (**e**) corresponding EDX spectra of RF and RM.

As can be seen in Figure 1a, a heterogeneous photo-self-Fenton degradation system was structured with RF as H_2_O_2_ production photocatalyst and RM as iron source and H_2_O_2_ activating agent to purify the antibiotic wastewater. The crystal structure and crystallographic properties of RF and RM were characterized by X-ray diffraction (XRD) analysis, as exhibited in Figure 1b. For the RF sample, an extensive diffraction peak was observed around 2θ = 20° (*d* = 4.4 Å), which can be attributed to the (002) crystal plane of graphite carbon, confirming the presence of the π-stacked aromatics [22]. RM is a kind of industrial waste material rich in Fe_2_O_3_ and Al_2_O_3,_ produced during the process of preparing aluminum in aluminum plants, which is obtained using Bayer’s method [23]. The exact chemical composition was analyzed by X-ray fluorescence (XRF) (as provided in Table S1), which indicated that the main components of the RM were largely metal oxides, silicate, and a small number of other impurities, and among them, the contents of Fe_2_O_3_ and Al_2_O_3_ account to 49.81% and 24.18%, respectively. As presented in the XRD profile of RM, the 2θ values of 24.1, 33.1, 35.7, 41.0, 49.5, 54.1, 62.6, and 64.1° agree with the standard card of Fe_2_O_3_ (JCPDS: 79-1741), corresponding to the (012), (104), (110), (006), (113), and (202) crystal planes [24], which indicates Fe_2_O_3_ is the main component of the RM, which is consistent with the XRF results. Significantly, no XRD diffraction peaks of other metal oxides (i.e., Al_2_O_3_ and TiO_2_) were detected in the RM due to the amorphous state, low concentration, and high dispersion of these metal oxides [25]. In the Fourier transform infrared (FT-IR) spectra in Figure 1c, the RF resins displayed individual bands stemming from resorcinols, linkers, and residual groups, which fully confirmed the precise synthesis of RF [26]. The broad peak at 2900~3300 cm^−1^ is the stretching vibration of resorcinol, and the peak at 1650 cm^−1^ belongs to the stretching vibration mode of C=O, which is due to hydrothermal treatment at high temperature, which can produce quinoid groups [27]. Simultaneously, the sharp peaks at 1618 and 1400 cm^−1^ are caused by the C=C vibrations of the aromatic ring and the C-H vibration of the methylene linker, while the broad peak in the region of 1100~1300 cm^−1^ is attributed to the C-O vibration of resorcinol and methylol [28]. Similarly, it was observed that the broad peak at 2900~3300 cm^−1^ is the stretching vibration of the O-H bond, while the strong peak at 971 cm^−1^ is the characteristic vibration of the Fe-O bond, which is attributed to the presence of Fe_2_O_3_; the broad peak in the range of 500 to 750 cm^−1^ is the telescopic vibration peak of the Si-O bond, which was clearly displayed in the FT-IR spectra of RM [29]. Scanning electron microscopy (SEM) was employed to visualize the microstructure of RF and RM, as exhibited in Figure 1d,e. The morphology of the RF expressed regular spherical shapes with a diameter of about 500 nm, with negligible inhomogeneous spherical particles on the sphere, which may be attributed to incomplete polymerization resulting from small differences in the temperature during the high-temperature hydrothermal process [30]. In addition, the RM was a mixture including metal oxides and silicates, with its morphology presenting irregularly sized lumps and granules which were loosely arranged and shared large cracks and bifurcated structures on the surface [31]. The corresponding energy dispersion X-ray spectrum (EDS, Figure 1e) and element mapping images (Figures S1 and S2) of RM and RF suggest that the RF was mainly composed of C and O elements, while the RM possessed O elements and abundant Fe elements, which can provide cheap iron sources to create the conditions for the photo-self-Fenton system.

**Figure 2:**
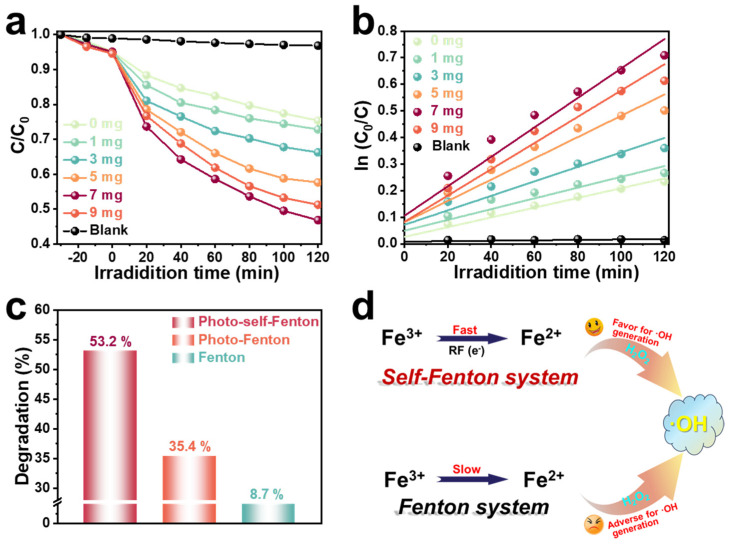
(**a**) Photo-self-Fenton TC degradation curves and (**b**) reaction kinetic fitting plots of RF/RM system under visible light irradiation. (**c**) Comparison of the degradation performance of RF/RM photo-self-Fenton system, photo-Fenton system and RM-based Fenton system (Addition of H_2_O_2_: 500 μM). (**d**) Simple mechanism diagram in photo-self-Fenton and Fenton systems.

The degradation performance of the heterogeneous RM/RF photo-self-Fenton system was estimated via tetracycline (TC) degradation under visible light (λ > 400 nm) illumination. The concentration of Fe species is one of the prerequisites for turning on the Fenton reaction [32], so the effect of different amounts of RM on the TC degradation performance of RF-based photo-self-Fenton system was explored (Figure 2a). As shown in Figure S3, the adsorption equilibrium was reached by adsorption after 30 min in the dark, excluding the interference of adsorption to the catalytic reaction. It is clearly observed that the degradation performance was improved significantly with the addition of RM, and the degradation performance reached the optimum and the degradation rate reached 53.2% with the addition of 7 mg of RM, which was due to the efficient utilization of H_2_O_2_ promoted by the activation by RM. Nevertheless, the employ of excess iron source would similarly inhibit the degradation of TC, as the excess RM could agglomerate, thereby blocking the light absorption of RF during stirring [33,34]. Furthermore, the corresponding pseudo-first order kinetic curves are displayed in Figure 2b; regarding the fitted degradation, the apparent reaction rate constants of *k* (min^−1^) are given in Table S2. Similarly, the kinetic reaction rate of the photo-self-Fenton degradation system with the addition of 7 mg of RM achieved a distinct increase of about 3.03 times compared to that of the pure RF photocatalytic system, which was consistent with the above results. Notably, it was crucial to determine whether the improved degradation performance stemmed from the material’s photocatalytic properties or whether it was acting as an H_2_O_2_ activator of RM in the self-Fenton system, since RM is a collection of various semiconductor materials. As provided in Figure S4, the TC degradation performance of RM with/without adding RF was compared under the photo-reaction conditions, and it was discovered that the increase of the photocatalytic degradation rate of pure RM was much less than that of the photocatalytic self-Fenton system after the addition of 20 mg RF with the increase of RM, which can fully confirm the role of RM as photocatalysts and H_2_O_2_ activators in the photocatalytic self-Fenton system. Meanwhile, the TC degradation performance was contrasted with common Fenton, photo-Fenton and photo-self-Fenton systems, as shown in Figure 2c, and it was concluded that the photo-self-Fenton system possessed the best degradation efficiency of TC under visible light irradiation, which is 1.52 and 6.11 times that of Fenton and photo-Fenton systems, respectively. Compared with the Fenton reaction, the cause of the enhanced performance of the self-Fenton reaction is the faster valence state circulation of iron ions to further affect the rate of hydroxy radical (^•^OH) formation of the major degradative active species (Figure 2d) [35], which is ascribed to the following reasons: (i) compared with ordinary Fenton systems, photo-self-Fenton systems employ semiconductor materials as the catalyst, which are excited by the external light source to produce photo-induced electrons, and thus quickly realize the valence state transition to activate H_2_O_2_ to produce ^•^OH [36]; (ii) simultaneously, electrons participate in the formation of H_2_O_2_ intermediates and provide raw materials for iron ion conversion [37]; (iii) in contrast, although both use the assistance of photocatalysis, the in situ generation of H_2_O_2_ in self-Fenton system increases the H_2_O_2_ content in the fixed environment, thus facilitating the valence cycle and H_2_O_2_ activation to produce highly oxidized ^•^OH, compared with photo-Fenton systems.

**Figure 3:**
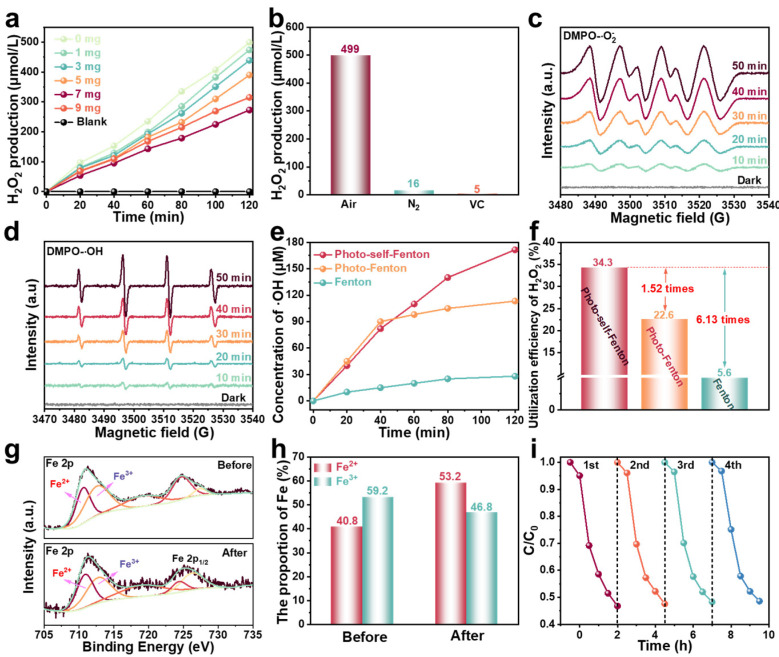
(**a**) Concentration of H_2_O_2_ produced by RF/RM photo-self-Fenton systems with different amounts of RM under visible light irradiation. (**b**) H_2_O_2_ production of RF under controlled experimental conditions. (**c**) ESR spectra of (**c**) DMPO-^•^O_2_^−^ and (**d**) DMPO-^•^OH on RF/RM photo-self-Fenton system under different irradiation time. (**e**) Variation curves of ^•^OH radial concentration and (**f**) utilization efficiency comparison of H_2_O_2_ over Fenton, photo-Fenton, and photo-self-Fenton systems. (**g**) High-resolution XPS spectra of Fe 2p and (**h**) the corresponding proportion of Fe for the RM before and after photocatalysis–self-Fenton degradation reaction. (**i**) Reusability of RF/RM photo-self-Fenton system.

From the analysis of the above results, the RF-based photo-self-Fenton system possessed a strong degradation performance, which stemmed from the synergy of photocatalysis and the Fenton method, so as to effectively generate and utilize H_2_O_2_, effectively promoting the separation of photo-induced carriers and effective conversion to ^•^OH [38,39]. RF is a metal-free semiconductor with a suitable band structure (Figure S5) and is endowed with a strong ability to produce H_2_O_2_ [40]. Consequently, the H_2_O_2_ production performance of the photo-self-Fenton system supplemented with different contents of RM was evaluated. As seen in Figure 3a, the production of H_2_O_2_ from RF was up to 499 μM within 2 h of visible light irradiation, based the standard curves for photocatalytic H_2_O_2_ production (Figure S6), and gradually decreased with the addition of RM due to the activation of H_2_O_2_ by RM. Generally, the formation of H_2_O_2_ follows two routes with two involved electrons: the water oxidation reaction (WOR) and the oxygen reduction reaction (ORR) [41]. Different from the WOR process of direct two electrons in one step, the process of ORR can be divided into one-step two-electron (O_2_ + 2e^−^ + 2H^+^ → H_2_O_2_) process and the two-step single-electron (O_2_ + e^−^ → ^•^O_2_^−^, ^•^O_2_^−^ + e^−^ + 2H^+^ → H_2_O_2_) process [42]. Therefore, the formation of H_2_O_2_ was deeply explored via changing the testing conditions, and the results are provided in Figure 3b. First, the H_2_O_2_ production of an as-prepared sample was tested under an N_2_ atmosphere, and it was discovered that only trace amounts of H_2_O_2_ were produced in the system with the discharge of O_2_, which directly excluded the function of WOR reaction and indicated that the H_2_O_2_ in the photo-self-Fenton system was derived from the ORR pathway. Afterwards, the production of H_2_O_2_ dropped sharply after superoxide radicals (^•^O_2_^−^) were captured by vitamin C (VC), indicating that a two-step single-electron ORR involving a radical intermediate remained to generate H_2_O_2_ in the system. As exhibited in Figure 3c, electron spin resonance (ESR) spectra were measured to detect ^•^O_2_^−^ with 5,5-dimethyl-1-pyrroline N-oxide (DMPO) during the photo-self-Fenton degradation of TC. There was no obvious characteristic signal of DMPO-^•^O_2_^−^ under darkness, while the signals of DMPO-^•^O_2_^−^ could be surveyed after 10 min of visible light irradiation and gradually became stronger within 50 min, revealing that the production of ^•^O_2_^−^ was continuous and stable, which favored the generation of H_2_O_2_ by reacting with electrons and was further activated by RM to produce ^•^OH in the self-Fenton system. Similarly, the ESR signals of ^•^OH were also carried out (Figure 3d), and the signal intensity of DMPO-^•^OH gradually became more significant, which fully established that ^•^OH are generated by H_2_O_2_ activation and are positively correlated with H_2_O_2_ production in the presence of an adequate iron source. The concentration of ^•^OH in the Fenton, photo-Fenton and photo-self-Fenton systems was further monitored by detecting the PL signal intensity of 7-hydroxycoumarin (Figure S7), where coumarin was used as a trapping agent for ^•^OH because of the blue fluorescence of 7-hydroxycoumarin generated by the ^•^OH and coumarin reaction [43]. As exhibited in Figure 3e, after 2 h of reaction, the accumulated ^•^OH concentration was approximately 28 μM in the Fenton system, while the concentration of ^•^OH reached 113.3 μΜ within 40 min, which did not significantly increase over time in the photo-Fenton system. Impressively, ^•^OH could be continuously and steadily generated in the photocatalytic–Fenton system; thus the ^•^OH concentration reached 171.5 μM and maintained a sustained uptrend over 2 h of reaction time. In addition, the utilization efficiencies of H_2_O_2_ (^•^OH transformation efficiencies) of different systems were calculated, and the related results were provided in Figure 3f. The ^•^OH transformation efficiency of the photo-self-Fenton reached 34.3%, approximately 1.52 and 6.13 times compared with that of the photo-Fenton and Fenton system, respectively, which may be intrinsic to the higher degradation efficiency of the photo-self-Fenton system [44,45]. Apparently, the degradation efficiency of the photo-self-Fenton system based on RF originated from the in situ efficient production and superior utilization efficiency of H_2_O_2_. Fe^2+^ is the key to turning on Fenton-like reactions, due to its function in activating H_2_O_2_ to ^•^OH. As revealed in Figure 3g,h, X-ray photoelectron spectroscopy (XPS) of RM before and after the degradation reaction was performed. Compared with the Fe^3+^/Fe^2+^ ratio before the reaction (1.45), the value decreased to 0.88 after the reaction, confirming the reduction of Fe^3+^ to Fe^2+^ during the self-Fenton degradation reaction [46]. Moreover, the RF maintained stable degradation activity after 4 cycles (Figure 3i), and the crystal structure, chemical composition, optical properties (Figure S8) and micromorphology (Figure S9) of RF expressed no change after 4 cycles of degradation reactions, which confirmed the high stability of the RF photocatalytic material and the degradation of the RF-based photo-self-Fenton system.

**Figure 4:**
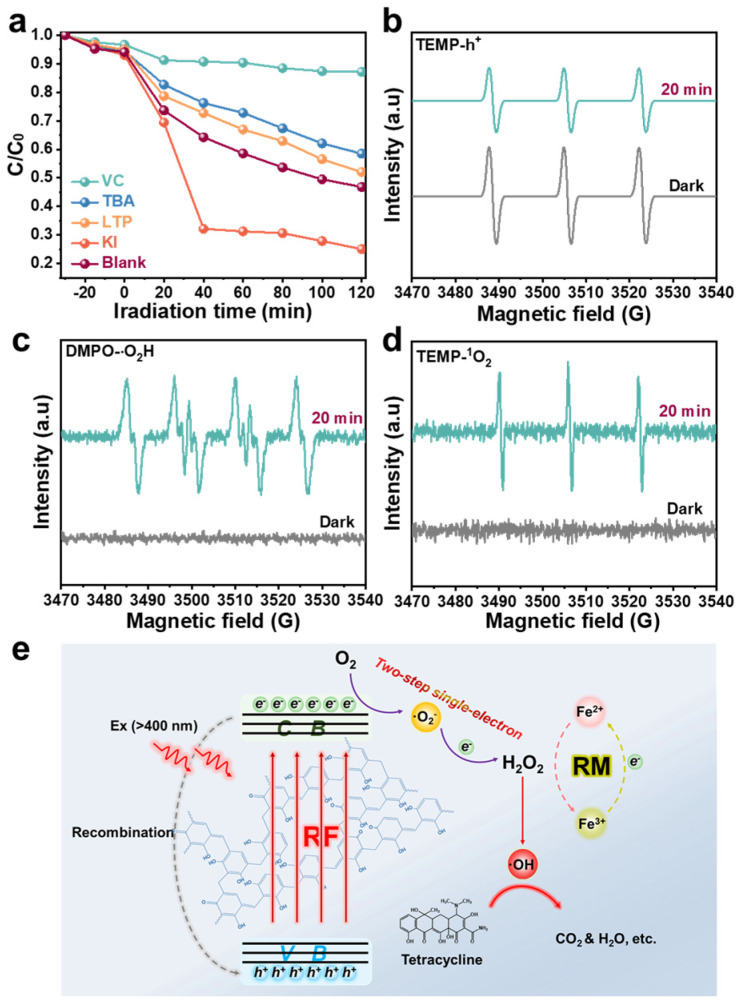
(**a**) Active species trapping experiment curves during the photo-self-Fenton degradation of TC over the RF/RM photo-self-Fenton system. ESR spectra of (**b**) DMPO-*h^+^*, (**c**) DMPO-O_2_H and (**d**) TEMP-^1^O_2_ for RF/RM photo-self-Fenton system. (**e**) Possible degradation mechanism of RF/RM photo-self-Fenton system.

In general, the photocatalytic degradation performance relies on the varieties and amount of active free radicals available during the degradation process, such as ^•^O_2_^−^, ^•^OH, singlet oxygen (^1^O_2_), and holes (*h^+^*) [47]. To further comprehend the mechanism of the TC degradation process, the free radical capturing experiments were carried out on the degradation system via adding vitamin C (VC), tert-butanol (TBA), L-tryptophan (LTP), and potassium iodide (KI) as capture ^•^O_2_^−^, ^•^OH, ^1^O_2,_ and *h^+^*. As exhibited in Figure 4a and Figure S10, the final degradation rates of TC were effectively suppressed by 12.85% and 41.54% after the addition of VC and TBA, respectively, which indicates that ^•^O_2_^−^ and ^•^OH are the main active free radicals in the RF/RM photo-self-Fenton system. Unlike the strong oxidation function of ^•^OH, ^•^O_2_^−^ acts as an intermediate of H_2_O_2_ and suppresses the degradation efficiency by inhibiting H_2_O_2_ production. The degradation rate decreased slightly after the introduction of LTP, indicating that ^1^O_2_ is not the main reactive oxygen species (ROS) during the photo-Fenton process. Contrarily, the degradation efficiency, which increased significantly with *h^+^*, was captured by KI, indicating that the *h^+^* plays a role in inhibiting degradation in this photo-self-Fenton system, rather than participating in the ring-opening reaction of TC as an oxide species to enhance the degradation effect, which could be due to the obstruction of the valence transition of iron and the inhibition of H_2_O_2_ generation and transformation related to recombination of photo-generated carriers [48]. As a supplement to the free radical capturing experiments, ESR spectra have been measured to detect the ^•^O_2_^−^, ^•^OH, *h^+^*, ^•^O_2_H, and ^1^O_2_ with 5,5-dimethyl-1-pyrroline N-oxide (DMPO) and 2,2,6,6-tetramethyl-4-piperidine (TEMP) in this system [49], which are provided in Figure 3c,d and Figure 4b–d. Uniformly, there was no characteristic signal of DMPO-^•^OH, DMPO-^•^O_2_^−^, TEMP-^1^O_2_, or DMPO-^•^O_2_H in darkness, while after 20 min of visible irradiation, strong signals from DMPO-^•^OH, DMPO-^•^O_2_^−^, TEMP-^1^O_2_, and DMPO-^•^O_2_H could be observed, confirming the presence of ^•^OH,^•^O_2_^−^, ^1^O_2_, and ^•^O_2_H during the photo-self-Fenton degradation process of the RF-based heterogeneous system. The appearance of ^•^O_2_H confirms that the reduction of Fe^3+^is not only involved in obtaining electrons (e^−^ + Fe^3+^ → Fe^2+^), but also in the reduction reaction in H_2_O_2_ decomposition (Fe^3+^ + H_2_O_2_ → Fe^2+^ + ^•^O_2_H + H^+^, *k* = 0.001–0.01 L·mol^−1^ s^−1^), although this reaction is difficult and often ignored, owing to the slowness of the kinetic reaction. For the ESR spectra of *h^+^*, it was clearly observed that the signal of *h^+^* became weaker, thus illustrating the recombination behavior of the electron-hole pairs within 20 min. Accordingly, the possible reaction mechanism of the RF-based photo-self-Fenton degradation system can be deduced (Figure 4e). The first occurrence was the photocatalytic process that RF photocatalysts being excited to generate electron-hole pairs by visible light and further produce H_2_O_2_ via a two-step single-electron ORR (O_2_ + e^−^ → ^•^O_2_^−^, ^•^O_2_^−^ + e^−^ + 2H^+^ → H_2_O_2_) with the separated photo-electrons [50,51]. For the Fenton process, Fe^3+^ ions in the RM were reduced to Fe^2+^ ions via reacting with photo-electrons, and the formation of ^•^OH was further realized via activating H_2_O_2_ to degrade antibiotic target (TC) as well as the valence cycle of Fe ions (Fe^2+^ + H_2_O_2_ → Fe^3+^ + ^•^OH + OH^−^). Nevertheless, the position of VB for RF is more negative than the conversion potential between ^•^OH and H_2_O (2.4 eV), thus excluding the WOR path of ^•^OH (OH^−^ + *h^+^* → ^•^OH). The photo-self-Fenton system could be operated continuously through the cyclic reaction of H_2_O_2_ with Fe^2+^.

**Figure 5:**
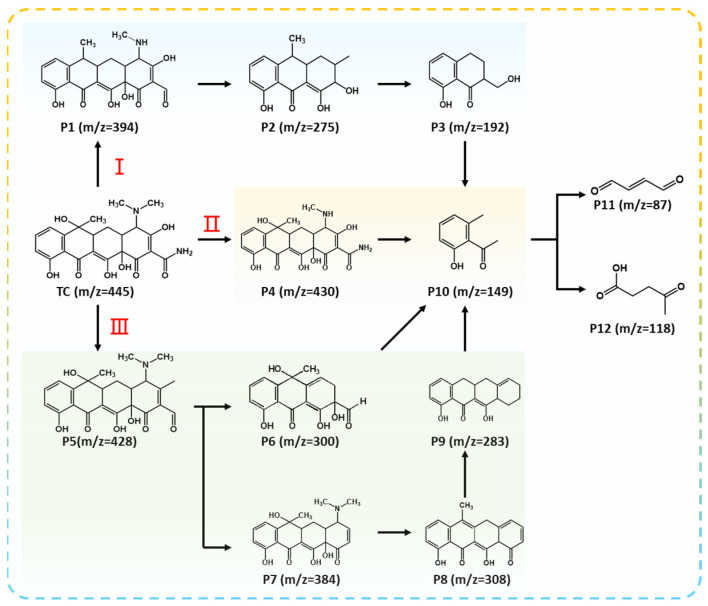
Possible degradation pathways and intermediates of RF/RM photo-self-Fenton system.

The major intermediates produced during the process of photocatalytic degradation for TC in RF/RM photo-self-Fenton system were further investigated via liquid chromatography–mass spectrometry (LC-MS), and the homologous spectra are displayed in Figure S11. The signals at 445 *m*/*z* are attributed to the TC molecular (C_22_H_24_N_2_O_8_) gradually decreasing with the degradation process, and other signals, imputed to smaller molecular compounds, were formed, indicating that TC was easily decomposed, catalyzed by the RF/RM photo-self-Fenton system. As shown in previous studies [52], intermediates are mainly produced through two modes: the loss or cleavage of specific functional groups and ring-opening reactions. Three possible degradation pathways of TC are thus proposed and exhibited in Figure 5. For pathway I, TC molecule breaks N–CH_3_ and C–NH_2_ at the same time as it loses a water molecule, resulting in product 1 (P1, *m*/*z* = 394). P1 is converted into P2 (*m*/*z* = 275) via ring opening reaction. Afterwards, P2 (*m*/*z* = 275) loses its methyl group and undergoes a continuous open-ring reaction to generate P3 (*m*/*z* = 192) and P10 (*m*/*z* = 149). For pathway II, P4 (*m*/*z* = 430) can be formed by losing a N-methyl in TC molecular. Then, P10 (*m*/*z* =149) is formed via demethylation and the breakage of the carbon–carbon single bond [53]. For pathway III, P5 (*m*/*z* = 428) can be formed by ^•^OH attack via dihydroxylation and N-demethylation, owing to the low energy of the N-C bond. Then, the next degradation steps can be divided into two pathways. One is that P6 (*m*/*z* = 300) and P10 (*m*/*z* = 149) are formed through dihydroxylation, demethylation reaction, and benzene rings opening [54]. The other mode is consecutive dihydroxylation and demethylation resulting in the production of the intermediates P7 (*m*/*z* = 384), P8 (*m*/*z* = 308), and P9 (*m*/*z* = 283). Then, smaller molecules with an *m*/*z* of 149 are produced during the ring-opening reactions’ progress. Finally, biodegradable intermediates with smaller molecules of P11 (*m*/*z* = 85) and P12 (*m*/*z* = 118) are generated through additional reactions, including dihydroxylation, decarboxylation, and benzene ring-opening reactions [55]. Ultimately, the 12 degradation intermediates of TC decomposition are detected using LC-MS, and a part of above intermediate could be completely decomposed into CO_2_ and H_2_O by the collaborative efforts of active species [56,57].

## 3. Experimental Section

### 3.1. Materials

Tetracycline (C_22_H_24_N_2_O_8_), resorcinol (C_6_H_6_O_2_, AR), formaldehyde solution (CH_2_O, 37 wt.%), and ammonium hydroxide solution (NH_3_•H_2_O, 28 wt.%~30 wt.%) were purchased from Macklin Biochemical Co., Ltd. (Shanghai, China) Red mud (RM) powder was obtained via Bayer’s method and provided by Chongqing Nanchuan District Pioneer Alumina Co., Ltd. (Chongqing, China) Prior to use, RM powder was first dried in an oven at 110 °C, and then the pretreated RM solid powder was screened by 175 mesh screens.

### 3.2. Preparation of Resorcinol–Formaldehyde (RF) Resin

The RF resin was prepared following the typical hydrothermal method reported previously [58]. In brief, 792 mg of C_6_H_6_O_2_, 1080 μL CH_2_O solution, and 460 μL NH_3_•H_2_O were added to 60 mL of deionized water, and a white colloidal suspension was obtained by stirring at room temperature. Subsequently, the above as-prepared suspension was transferred to a stainless-steel autoclave and heated at 250 °C for 24 h in an oven. After cooling to room temperature, the resulting RF resin solids were thoroughly washed with water and ethanol and further dried under vacuum at 60 °C for 12 h.

### 3.3. Photo-Self-Fenton Degradation Experiments 

The photo-self-Fenton degradation efficiency of the organic contaminant (tetracycline (TC)) was evaluated under visible light irradiation (λ > 400 nm) using the multi-channels photoreactor (Pefectlight PCX-50C, Beijing Perfectlight Technology Co., Beijing, China). First, 20 mg RF and RM with different masses (m = 0, 1, 3, 5, 7 and 9 mg) were poured into quartz beakers containing 50 mL TC solution (20 mg/L) and stirred in darkness for 30 min to reach adsorption–desorption equilibrium. Subsequently, the overall system was then irradiated under visible light for 120 min, and 3 mL of the suspension was extracted and centrifuged at 20 min intervals and measured by UV-vis spectrophotometer at the characteristic absorption peak at the wavelength of 350 nm.

In addition, corresponding characterizations, detailed experimental procedures for photo-electrochemical tests, photocatalytic H_2_O_2_ production experiments, and the concentration and variation efficiency of ^•^OH radials and active free radical-capturing experiments are provided in the Supplementary Materials.

## 4. Conclusions

In summary, an emerging heterogeneous photo-self-Fenton system, constructed with RF resin as photocatalyst and RM industrial waste as activating agent of H_2_O_2,_ was proposed, which was supplied to treat antibiotic contamination in wastewater without the requirement of consumable chemical oxidants or high levels of energy. Compared with the photo-Fenton degradation performance of RM, the kinetic reaction rate of the optimal photo-self-Fenton degradation system achieved a distinct increase by 3.03 times under visible light irradiation. The photo-self-Fenton system in this paper possesses superiorities in at least three aspects: (i) inspired by the idea of turning waste into a treasure, the industrial waste RM was employed as an additional iron source to activate H_2_O_2_ to produce active species with strong oxidation, which theoretically triggers the enhanced degradation properties of the Fenton system as well as control of environmental pollution due to industrial production, killing two birds with one stone; (ii) in situ generation of H_2_O_2_ relieves pressure on operability, safety and costs, and this spontaneous formation of H_2_O_2_ is superior to exogenous H_2_O_2_, making the degradation of photo-self-Fenton systems far more effective than that of Fenton and photo-Fenton reactions; (iii) the RF/RM photo-self-Fenton system applies visible light as the energy injection source, avoiding the introduction of high-intensity energy fields (e.g., UV/chlorine ultraviolet methods and electrochemical methods) and achieves the sustainability of environmental governance. This study provides sustainable design ideas for the development of waste-based heterogeneous photocatalysis–self-Fenton for water environmental restoration.

## Data Availability

Samples of the compounds are available or not available from the authors.

## Supplementary Materials

The following supporting information can be downloaded at: https://www.mdpi.com/article/10.3390/molecules29071514/s1. Figure S1 HAADF-SEM and elemental mapping images of RF. Figure S2 HAADF-SEM and elemental mapping images of RM. Figure S3 (a) TC adsorption curves and (b) adsorption kinetic fitting plots of RF/RM system in dark condition. Figure S4 Comparison of the TC degradation performance of RM with/without adding RF under the photo-reaction condition. Figure S5 (a) UV-vis absorption spectrum and (b) *E_g_* plot of (*αhv*)^2^ presented by the Kubelka-Munk transformed reflectance diffuse spectrum of RF. (c) Mott-Schottky plots and (d) Energy band structure diagram of RF. Figure S6 (a) UV-vis absorption spectra of H_2_O_2_ solutions with different concentrations and (b) corresponding calibration curve for testing H_2_O_2_ production at the absorption peak of 350 nm. Figure S7 (a) PL spectra of 7-hydroxycoumarin with different concentrations and (b) corresponding calibration curve for testing 7-hydroxycoumarin concentrations. Figure S8 (a) XRD patterns, (b) FT-IR and (c) UV-vis absorption spectra of used RF sample after photocatalytic reaction. Figure S9 SEM image of used RF sample. Figure S10 Degradation rates of TC by adding different radical scavengers. Figure S11 LC-MS spectra of possible intermediates by the photocatalytic self-Fenton degradation process of TC over RF/RM photo-self-Fenton system. Table S1 Main chemical compositions of RM. Table S2 Kinetic constants for TC photo-self-Fenton degradation of RF/RM system under visible light irradiation.
