# Supplementary material for: Construction of a Visible-Light-Response Photocatalysis–Self-Fenton Degradation System of Coupling Industrial Waste Red Mud to Resorcinol–Formaldehyde Resin
Source: Molecules. 2024 Mar 28;29(7):1514. doi: 10.3390/molecules29071514 (PMC11013769; doi:10.3390/molecules29071514)
Supplement: Supplementary file 1 [file molecules-29-01514-s001.zip › molecules-2908529-supplementary.pdf]

Construction of a Visible-Light-Response Photocatalysis–Self-Fenton Degradation System of Coupling Industrial Waste Red Mud to Resorcinol–Formaldehyde Resin

Supporting Information

Xiangxiu Lv [1,†], Hao Yuan [1,†], Kaiqu Sun [1], Weilong Shi [1,*], Chunsheng Li [2,*] and Feng Guo [3,*]

[1] School of Material Science and Engineering, Jiangsu University of Science and Technology, Zhenjiang 212100, China
[2] Key Laboratory of Advanced Electrode Materials for Novel Solar Cells for Petroleum and Chemical Industry of China, School of Chemistry and Life Sciences, Suzhou University of Science and Technology, Suzhou 215009, China
[3] School of Energy and Power, Jiangsu University of Science and Technology, Zhenjiang 212100, China
* Correspondence: shiwl@just.edu.cn (W.S.); lichsheng@163.com (C.L.); gfeng0105@126.com (F.G.)
† The authors contributed equally to this work.

1. Characterizations

X-ray diffraction (XRD, Apex II, Bruker X-ray diffractometer) patterns were operated to characteristic the crystal phase of samples in the range of 2θ =10-80°. The Fourier transform infrared (FT-IR) spectra were tested by a Nicolet 6700 spectrometer in the range of 4000-500 cm^{-1}. The performed UV-Vis diffuse reflectance spectra (UV-Vis DRS) were record by Shimadzu UV-3100 spectrophotometer to detect the optical properties of the materials in the wavelength of 300 to 800 nm. The microscopic morphology, energy dispersive X-ray spectroscopy (EDS) measurement and element mapping of as-prepared samples was conducted on scanning electron microscope (SEM, Hitachi S-4800). The electron spin resonance (ESR) of free radicals was measured with a Bruker A300 spectrometer under visible light irradiation ($\lambda > 420$ nm). The composition of the red mud was characterized by the AxiosPW4400 X-ray fluorescence (XRF) pair of PANalytic B.V. The intermediate products were analyzed by liquid chromatography-mass spectrometry (LC-MS) (Ultimate 3000 UHPLC-Q Exactive). The photoluminescence (PL) spectra was monitored by Edinburgh FLS980 transient fluorescence spectrophotometer. X-ray photoelectron spectroscopy (XPS) was measured by VG Multilab 2000 instrument with Al Kα source to identify the chemical states.

2. Photoelectrochemical tests

The plot Mott-Schottky (MS) was measured by Chenhua CHI 660E with three-electrode system at the solution of Na_2SO_4 (0.5 M, pH= 6.8). 10 mg of samples and 20 μL of 0.5% naphthol solution were added into 1 mL deionized water before dispersion by ultrasound. The solution of 30 μL was deposited on fluorine-doped tin oxide (FTO) conductive glass (2 cm × 3 cm) 5 times in total. The FTO glass deposited with photocatalyst was dried under nitrogen at 60 ℃, after which the working electrode was prepared. The dried FTO glass, platinum plate and Ag/AgCl (containing saturated potassium chloride electrolyte) were used as working electrode, counting electrode and reference electrode, respectively, to constitute a three-electrode system. The calomel electrode system can be converted to a reversible hydrogen electrode (RHE) system by the equation: E(NHE)=E(Ag/AgCl) + 0.197.

3. Photocatalytic H_2O_2 production experiments

Photocatalytic H_2O_2 production experiments were carried out via a multi-channel photochemical reaction system (Beijing Pefectlight PCX-50C). The visible light source (λ > 400 nm) was provided by the lamp panel equipped with 10 W LED lamp. Briefly, 20 mg photocatalyst was initially added to 30 mL of deionized water to form a homogeneous suspension and exposure to the visible light irradiation. Subsequently, 2 mL of the suspension was taken out from the reaction cell at regular intervals and removed the photocatalyst powder by centrifugation. The concentration of H_2O_2 was then measured via the iodometric method. Typically, a volume of fluorescent reagents (0.1 M potassium hydrogen phthalate and 0.4 M potassium iodide in water) were complexed with the reaction system, I^- can react with H_2O_2 to form I^{3-}, which can be detected at 350 nm with a UV spectrometer ($H_2O_2 + 3I^- + 2H^+ = I_3^- + 2H_2O$), the absorbance at 350 nm is measured using a UV-Vis spectrophotometer after 30 min reaction period and combined with the measured standard curve to estimate the concentration of H_2O_2 during each reaction.

4. The concentration and variation efficiency of ·OH radials

The concentration and variation efficiency of ·OH radials were calculated according to the method reported by predecessors. Since the conversion rate of ·OH to 7-hydroxycoumarin is 6.1%, the intensity of 7-hydroxycoumarin is measured by fluorescence spectrometer, and then the corresponding concentration of 7-hydroxycoumarin is determined according to the linear equation. The variation efficiency of ·OH radials is generally considered as the utilization of H_2O_2 in the photocatalysis-self-Fenton reaction, which was calculated as follows:

$$\text{H}_2\text{O}_2 \text{ utilization efficiency (Photocatalysis-self-Fenton)} = \frac{\text{H}_2\text{O}_2 \text{ (Effective utilization)}}{\text{H}_2\text{O}_2 \text{ (Total)}}$$

5. Active free radicals capturing experiments

During active free radicals capturing experimental process, vitamin C (VC), tertiary butanol (TBA), L-tryptophan (LTP) and potassium iodide (KI) were served as scavengers to capture superoxide radicals ($\cdot O_2^-$), hydroxy radicals ($\cdot OH$), singlet oxygen ($^1O_2$) and holes (h^+), respectively. The details of capturing experiments are consistent with the photocatalytic degradation experiments except for the addition of 1 mmol scavengers before the experimental process.

6. Figures and Tables

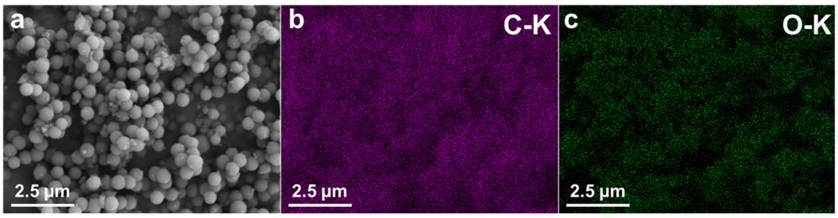

Figure S1 HAADF-SEM and elemental mapping images of RF.

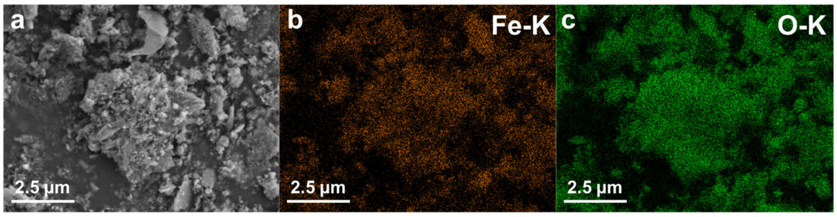

Figure S2 HAADF-SEM and elemental mapping images of RM.

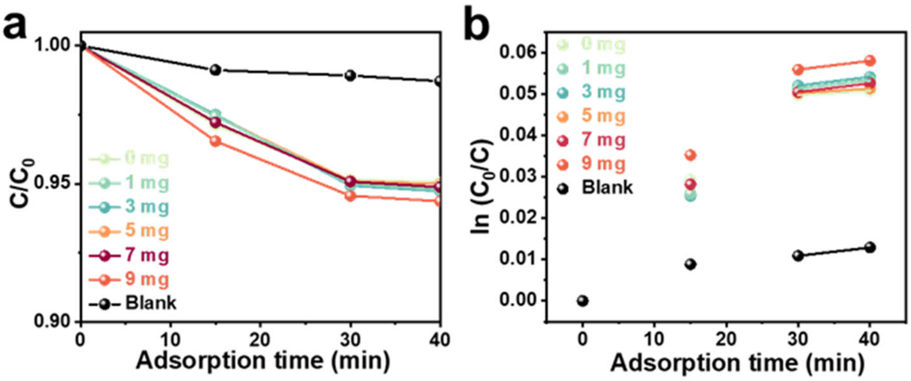

Figure S3 (a) TC adsorption curves and (b) adsorption kinetic fitting plots of RF/RM system in dark condition.

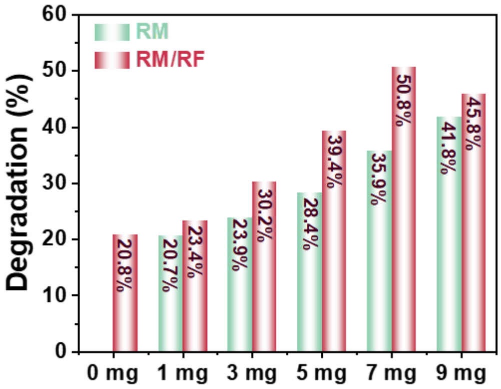

Figure S4 Comparison of the TC degradation performance of RM with/without adding RF under the photo-reaction condition.

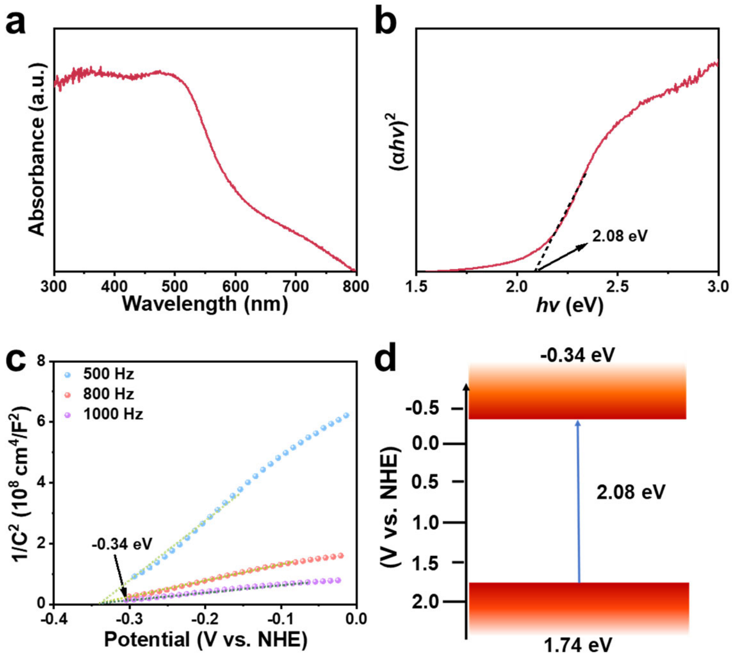

Figure S5 (a) UV-vis absorption spectrum and (b) E_g plot of $(\alpha hv)^2$ presented by the Kubelka-Munk transformed reflectance diffuse spectrum of RF. (c) Mott-Schottky plots and (d) Energy band structure diagram of RF.

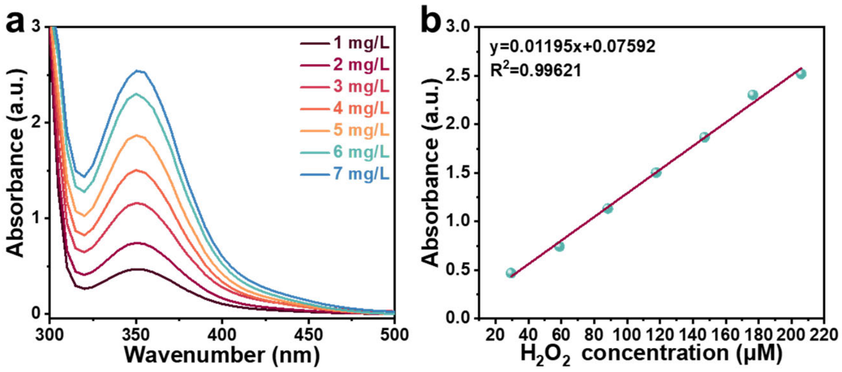

Figure S6 (a) UV-vis absorption spectra of H_2O_2 solutions with different concentrations and (b) corresponding calibration curve for testing H_2O_2 production at the absorption peak of 350 nm.

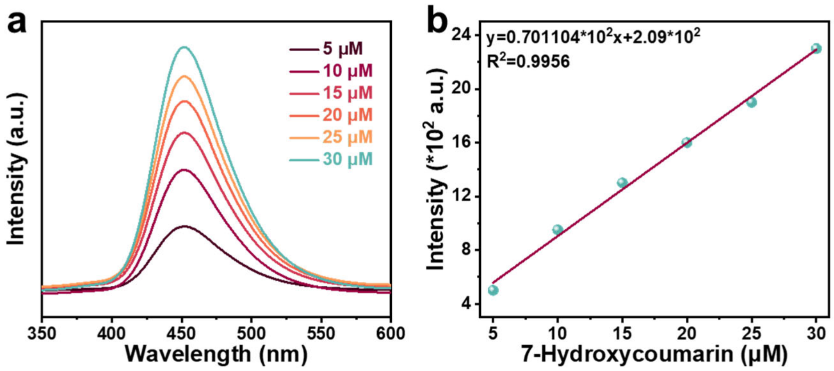

Figure S7 (a) PL spectra of 7-hydroxycoumarin with different concentrations and (b) corresponding calibration curve for testing 7-hydroxycoumarin concentrations.

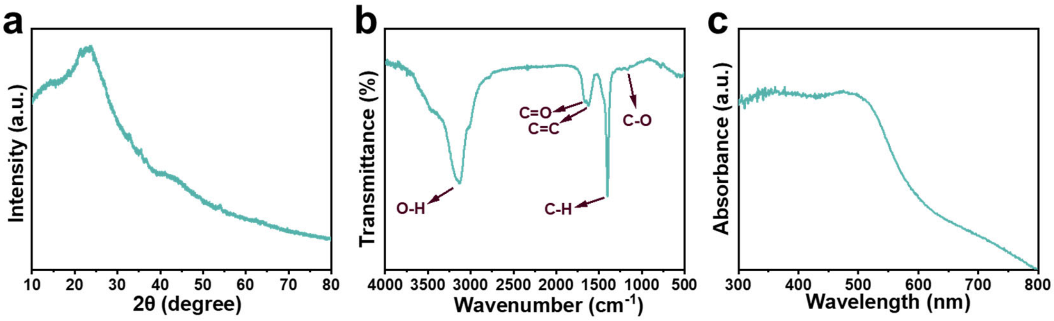

Figure S8 (a) XRD patterns, (b) FT-IR and (c) UV-vis absorption spectra of used RF sample after photocatalytic reaction.

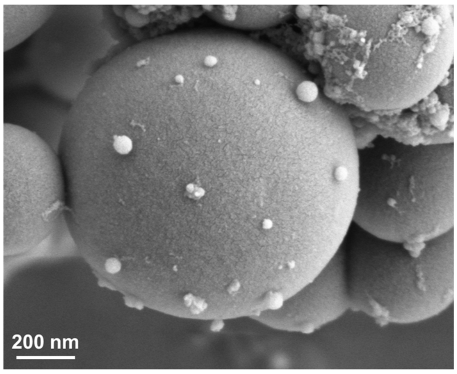

Figure S9 SEM image of used RF sample.

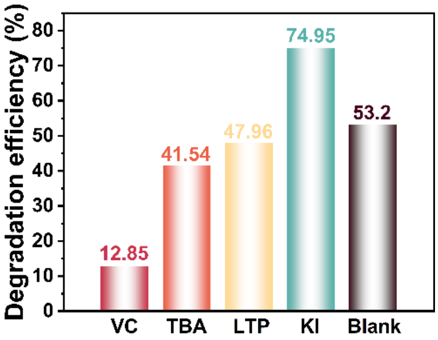

Figure S10 Degradation rates of TC by adding different radical scavengers.

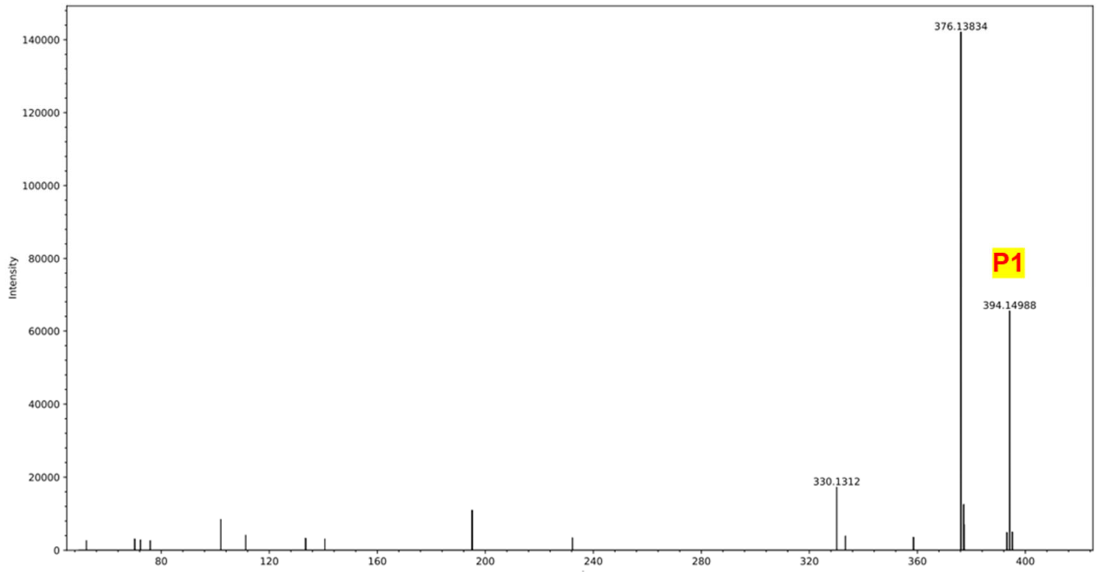
376.13834
P1
394.14988
330.1312
Intensity

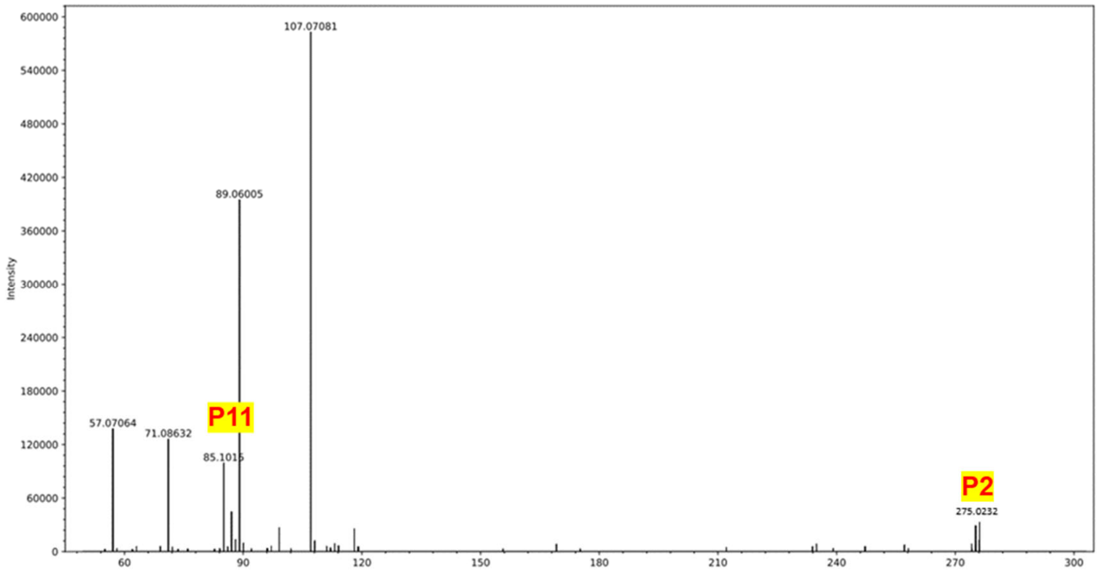
107.07081
89.06005
57.07064
71.08632
P11
85.1015
P2
275.0232
Intensity

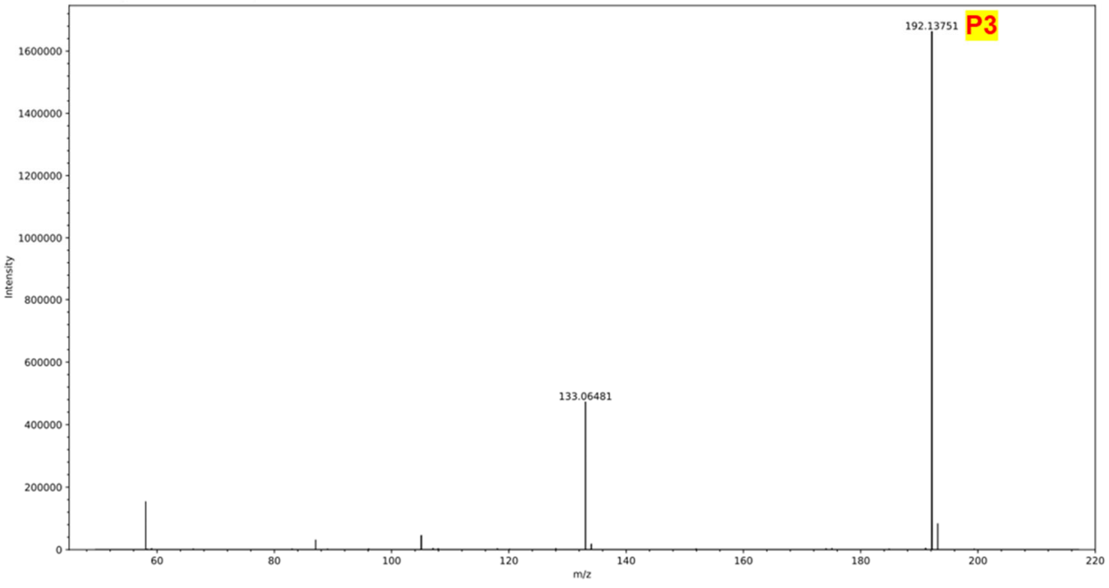
192.13751
P3
133.06481
Intensity
m/z

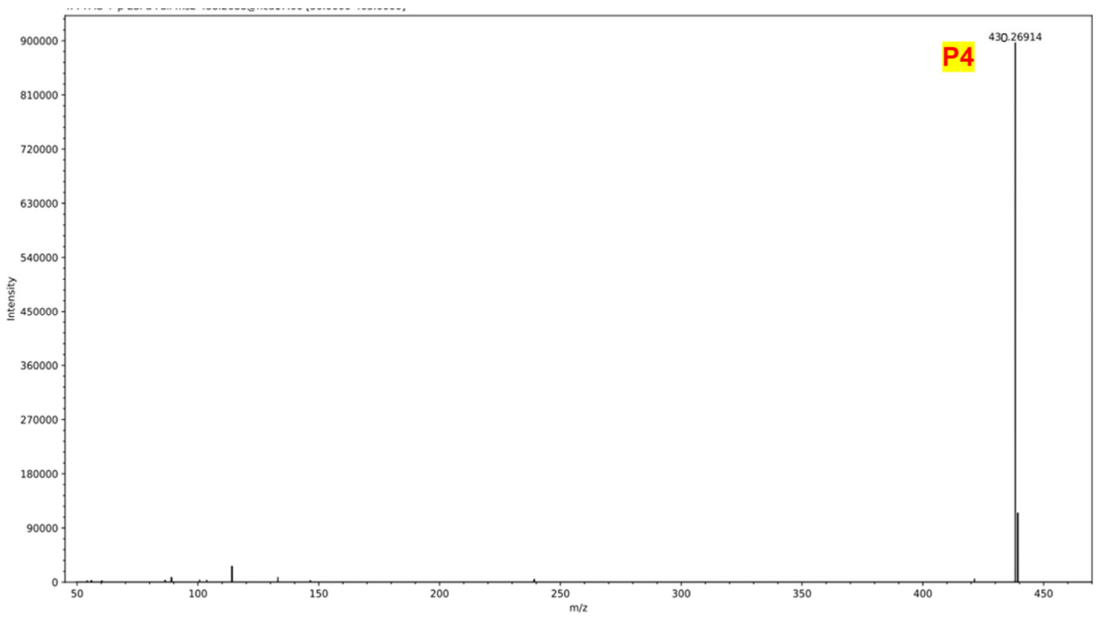
P4
430.26914
Intensity
m/z

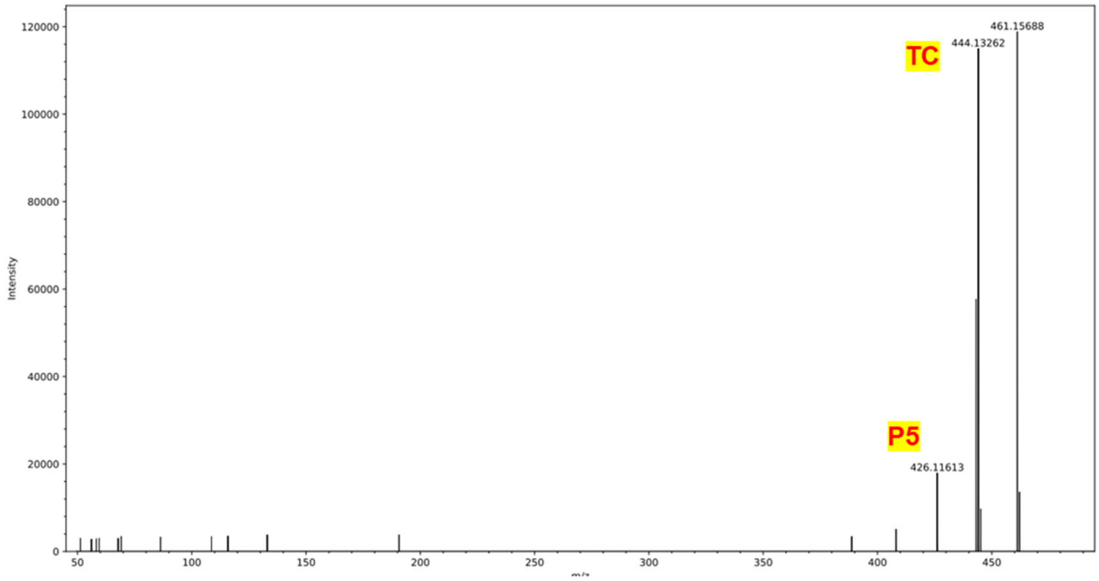
TC
444.13262
461.15688
P5
426.11613
Intensity

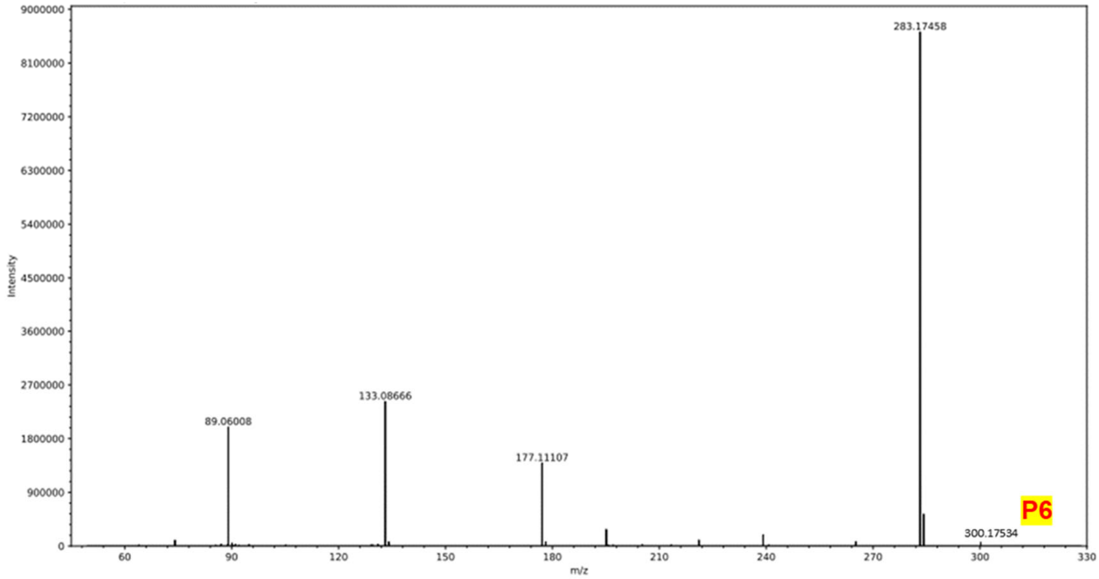
283.17458
133.08666
89.06008
177.11107
P6
300.17534
Intensity
m/z

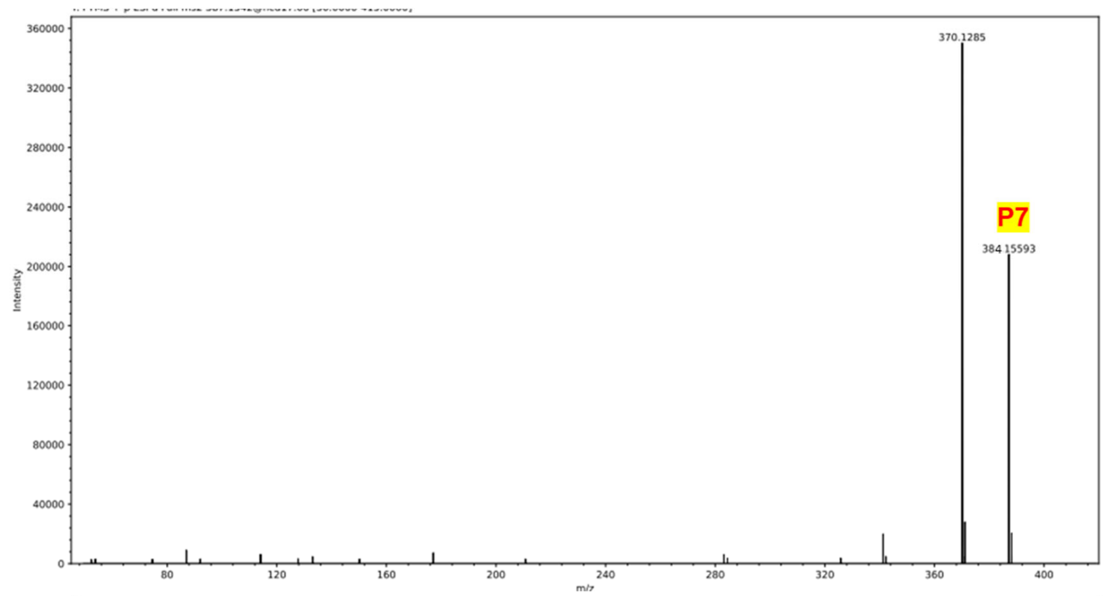
370.1285
P7
384.15593
Intensity
m/z

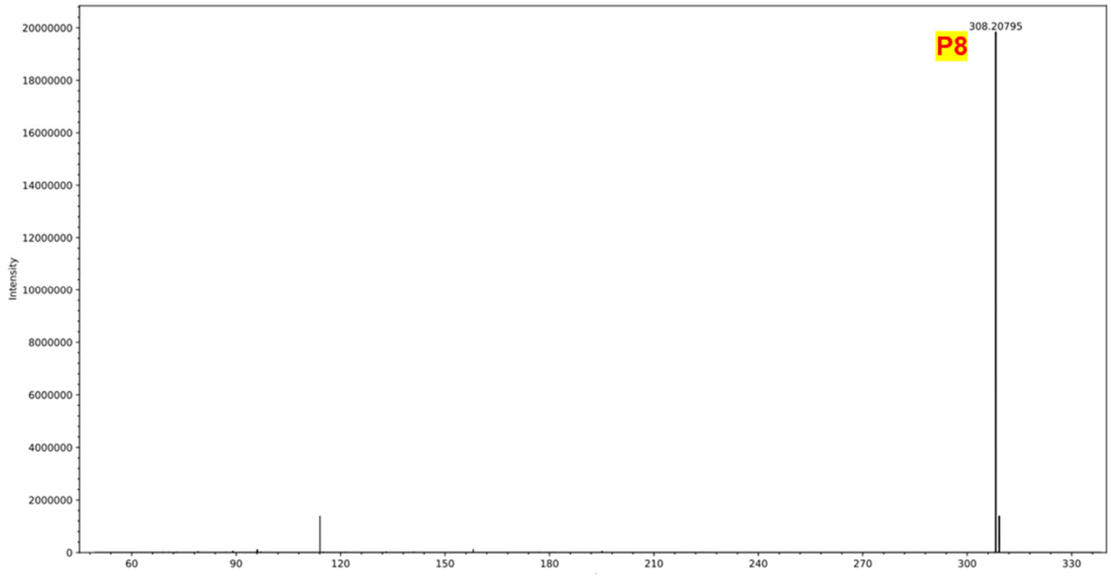
308.20795
P8
Intensity

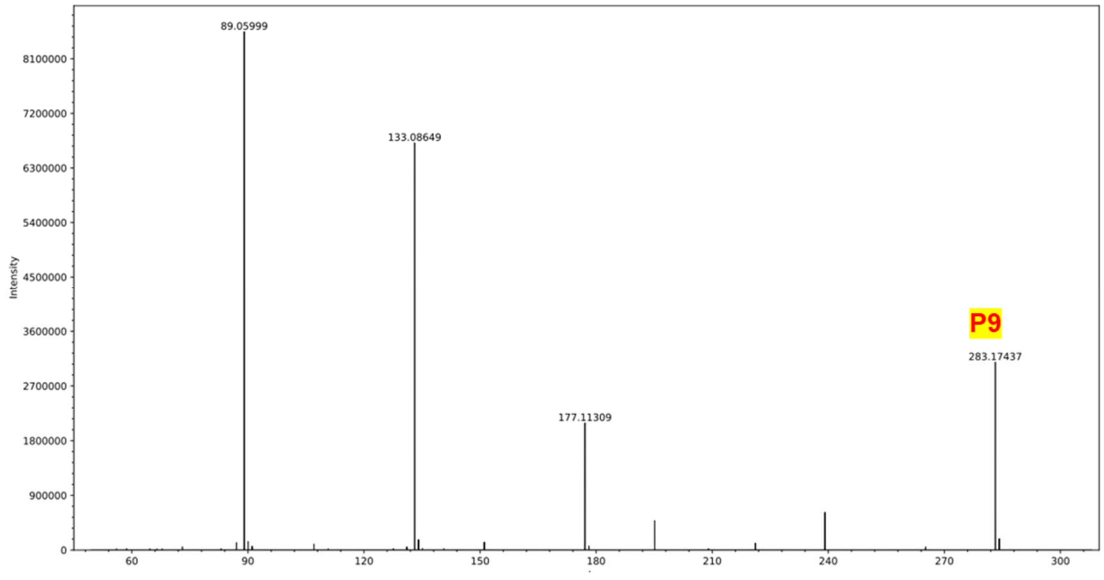
89.05999
133.08649
177.11309
P9
283.17437
Intensity

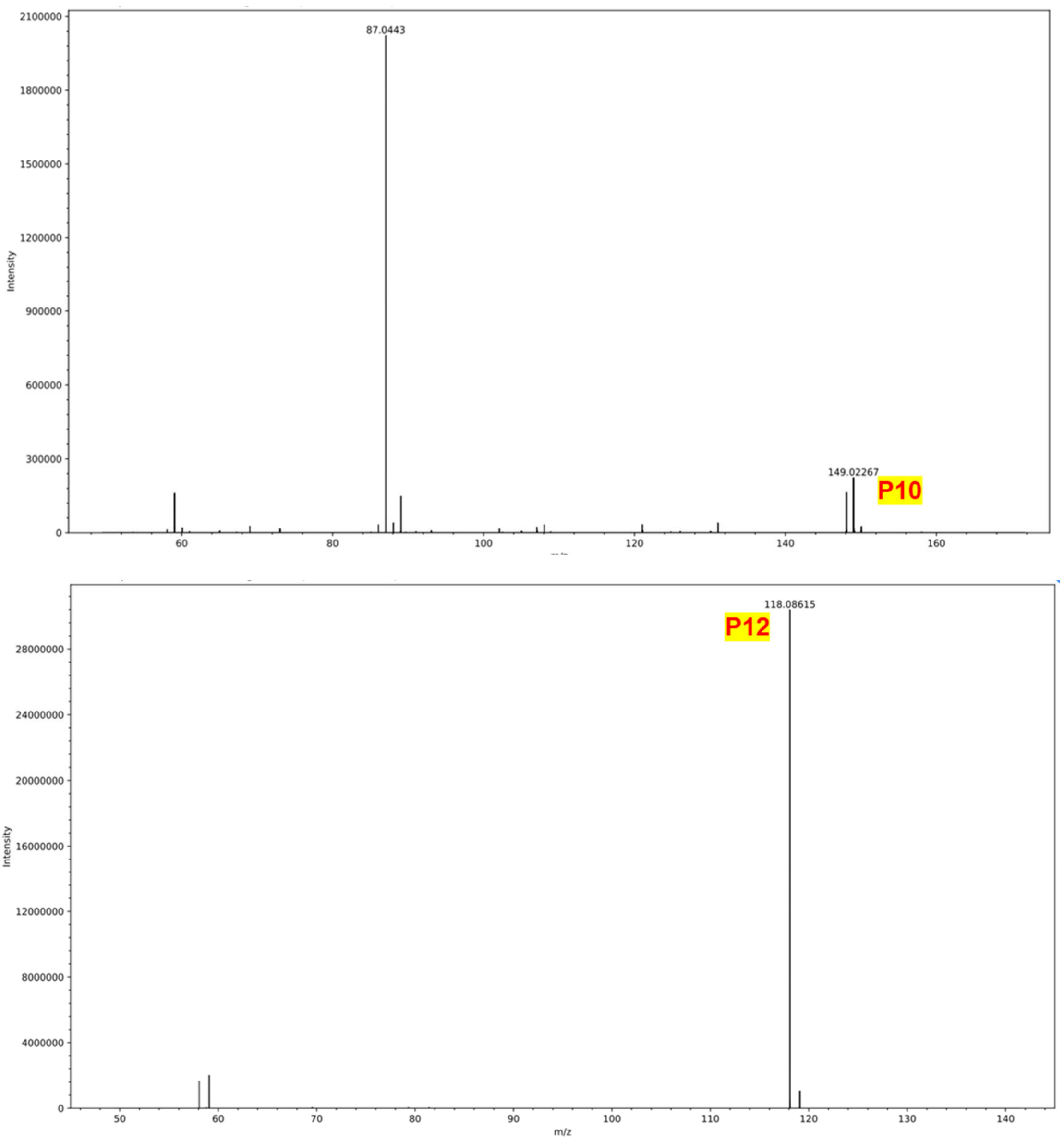

Figure S11 LC-MS spectra of possible intermediates by the photocatalytic self-Fenton degradation process of TC over RF/RM photo-self-Fenton system.

Table S1 Main chemical compositions of RM.

Chemical composition	Fe_2O_3	Al_2O_3	SiO_2	TiO_2	CaO	Na_2O	P_2O_5	others
**Mass content (wt)/%**	49.81	24.18	9.03	4.191	3.201	6.232	0.212	3.144

Table S2 Kinetic constants for TC photo-self-Fenton degradation of RF/RM system under visible light irradiation.

Amount of RM (mg)	k (min^{-1})
0 mg	0.00183
1 mg	0.00202
3 mg	0.00272
5 mg	0.004
7 mg	0.00554
9 mg	0.00494
